# Pharmacogenetics in Community Pharmacy: Global Perspectives and Implementation

**DOI:** 10.3390/jcm15093280

**Published:** 2026-04-25

**Authors:** Kinga Rutkowska, Beata Chełstowska, Urszula Religioni, Mariola Borowska, Adam Kobayashi, Regis Vaillancourt, Artur Białoszewski, Sebastian Sikorski, Zbigniew Doniec, Piotr Bromber, Agnieszka Biala, Krzysztof Kurek, Jakub Pawlikowski, Piotr Merks

**Affiliations:** 1 Department of Pharmacology and Clinical Pharmacology, Faculty of Medicine, Collegium Medicum, Cardinal Stefan Wyszyński University, 01-938 Warsaw, Poland; k.rutkowska@uksw.edu.pl (K.R.); a.kobayashi@uksw.edu.pl (A.K.); regisvaillancourt@hotmail.com (R.V.); s.sikorski@uksw.edu.pl (S.S.); p.merks@uksw.edu.pl (P.M.); 2 Department of Biochemistry and Laboratory Diagnostics, Faculty of Medicine, Collegium Medicum, Cardinal Stefan Wyszyński University, 01-938 Warsaw, Poland; b.chelstowska@uksw.edu.pl; 3 School of Public Health, Centre of Postgraduate Medical Education of Warsaw, 01-813 Warsaw, Poland; urszula.religioni@gmail.com (U.R.); piotr.bromber@cmkp.edu.pl (P.B.); 4 The Polish Pharmacy Practice Research Network (PPPRN), 00-867 Warsaw, Poland; bialaak@gmail.com; 5 Department of Epidemiology and Biostatistics, Medical University of Warsaw, 02-091 Warszawa, Poland; artur.bialoszewski@gmail.com; 6 Medical Institute, Academy of Applied Sciences, 34-400 Nowy Targ, Poland; zdoniec1@gmail.com; 7 University of Medical Sciences, 02-678 Warsaw, Poland; k.kurek@ppprn.org; 8 Department Social Medicine, Medical University of Lublin, 20-059 Lublin, Poland; jakub.pawlikowski@umlub.pl

**Keywords:** pharmacogenetics, pharmaceutical care, pharmacy

## Abstract

Pharmaceutical care provides the conceptual foundation for integrating pharmacogenetics into everyday pharmacy practice. Defined by Hepler and Strand as “the responsible provision of drug therapy for the purpose of achieving specific outcomes that improve a patient’s quality of life”, pharmaceutical care emphasizes a patient-centered approach in which the pharmacist collaborates with the patient, physician, and other healthcare professionals to design, implement, and monitor individualized therapeutic plans. In this context, pharmacogenetics can be regarded as an extension of pharmaceutical care: while the traditional model relies on monitoring patient outcomes and adherence, PGx adds a genetic dimension that allows treatment to be optimized from the very beginning. The pharmacist’s role therefore evolves from not only ensuring safe and effective use of medicines, but also interpreting genetic test results, supporting adherence to genetically guided therapy, and educating patients about the implications of their personal genetic profile. The introduction of pharmacogenetics testing as one of the potential services offered by community pharmacies is a promising proposition that may revolutionize the approach to drug therapy. Pharmacogenetics, a subset of pharmacogenomics, focuses on the study of DNA sequence variations that influence response to drugs. Thanks to advances in the field of genomics, it has become possible to study the genetic basis of variability in drug response. The identification of alleles responsible for the rapid or slow metabolism of xenobiotics has ushered in a new era in pharmacology. The aim of this interdisciplinary field, combining genetics and pharmacology, is to adapt treatment to a specific patient based on the analysis of their genome and gene polymorphism. Throughout the world, pharmacogenetics is gaining importance as a tool for personalizing medicine. In countries such as the United States, Canada, and the United Kingdom, programs integrating pharmacogenetics with healthcare are being developed. Clinical trials and the implementation of genetic tests into medical practice allow for better matching of medications and reducing the risk of side effects. Pharmacists will play a key role in integrating pharmacogenetics into healthcare. As specialists in the field of pharmacotherapy, they will support physicians in interpreting the results of genetic tests and adapting drug therapy to the individual needs of the patient. Additionally, pharmacists can educate patients and healthcare professionals about the benefits of pharmacogenetics and monitor the effects and safety of medications. Their involvement in the process of personalization of treatment may contribute to improving the effectiveness and safety of pharmacological therapies.

## 1. Introduction

Pharmacogenetics, as a key element of the emerging 4P medicine paradigm (personalized, preventive, predictive, and participatory), is rapidly transforming the landscape of drug therapy. By aligning treatment strategies with individual genetic profiles, pharmacogenetics offers the potential to enhance medication efficacy and safety, paving the way for a new era in personalized healthcare.

The European Medicine Agency (EMA, the European Union agency responsible for the scientific evaluation, supervision, and safety monitoring of drugs) defines pharmacogenomics as the study of DNA and RNA characteristics related to drug response. Pharmacogenetics is a subset of pharmacogenomics and is defined as ‘the study of variations in DNA sequence as related to drug response’ [1]. Pharmacogenetics and pharmacogenomics both study how genetic differences affect people’s responses to medicines, and both underpin personalized medicine. Pharmacogenetics usually refers to gene–drug relationships (e.g., CYP450 enzymes), often focusing on single genes affecting drug metabolism, while pharmacogenomics typically denotes a broader, genome-wide view of drug response. However, in most of the modern literature the terms are used interchangeably under the shared shorthand PGx.

Although both terms are often used interchangeably in the literature, the term pharmacogenetics (PGx) is used throughout this review for consistency. This review was conducted as a narrative synthesis to summarize and critically evaluate the current international evidence on the implementation of pharmacogenetics (PGx) in community pharmacy practice and its integration into clinical care. The objective was to identify and analyze existing initiatives, regulatory frameworks, and educational strategies supporting the incorporation of PGx into pharmacy-based patient care, with particular emphasis on implications for the Polish healthcare system.

## 2. Materials and Methods

A comprehensive literature search was conducted between January and March 2025 across six major electronic databases: MEDLINE (via PubMed), Embase, Scopus, Web of Science, the Cochrane Library, and Google Scholar. The search strategy combined free-text keywords and controlled vocabulary (MeSH and Emtree terms) related to pharmacogenetics and pharmacy practice. The following terms and their combinations were used: “pharmacogenetics,” “pharmacogenomics,” “genetic testing,” “personalized medicine,” “pharmacy practice,” “community pharmacy,” “clinical pharmacy,” “pharmacist,” “implementation,” “integration,” and “medication safety,” combined using Boolean operators (AND/OR). No date restrictions were applied, and only publications in English and Polish were included. Additional sources were identified through citation tracking and targeted searches of policy documents, implementation reports, and institutional guidelines (e.g., CPIC, DPWG, FDA, EMA, RNPGx).

All identified records were imported into EndNote X9 for organization and duplicate removal. Two reviewers independently screened titles and abstracts, followed by full-text assessment of potentially relevant articles. Discrepancies were resolved through discussion. Inclusion criteria encompassed original studies, reviews, reports, and guidelines addressing the implementation, clinical relevance, or system-level integration of pharmacogenetics, particularly in relation to pharmacy practice. Studies limited to laboratory or methodological genetics without clinical application, as well as editorials and non-evidence-based opinion papers, were excluded.

Given the narrative nature of this review, no formal quality assessment of included studies was performed. Instead, emphasis was placed on identifying key themes and patterns across the literature. Data were synthesized using a structured thematic approach, combining both a priori domains (regulatory frameworks, implementation models, clinical outcomes, pharmacoeconomic evidence, and education/competencies) and themes emerging from the literature (e.g., implementation barriers, data integration challenges, and variability in reimbursement models). Countries were selected for detailed analysis based on the availability of published implementation initiatives, relevance to community pharmacy practice, and diversity of healthcare system models.

To enhance transparency and reproducibility, the narrative synthesis followed a structured multi-step approach. First, all included studies were reviewed in full text and relevant information was extracted into predefined categories, including study design, clinical context, type of PGx intervention, implementation setting (e.g., community pharmacy, hospital), and reported outcomes. Second, extracted data were coded and grouped into thematic domains based on both a priori categories (regulatory frameworks, implementation models, clinical outcomes, pharmacoeconomic evidence, and education/competencies) and inductively identified themes emerging from the literature, such as implementation barriers, data integration challenges, and variability in reimbursement models. Thematic organization was conducted iteratively by the authors through discussion and consensus.

Given the narrative nature of this review, a formal risk-of-bias or quality assessment using standardized tools was not performed. However, efforts were made to prioritize higher-quality evidence, including systematic reviews, clinical studies, and official guidelines, and to critically interpret findings in light of study design and potential limitations.

Countries included in the comparative analysis were selected based on predefined criteria, including: (1) availability of published evidence on PGx implementation, (2) presence of national or regional initiatives relevant to pharmacy practice, (3) diversity of healthcare system models, and (4) relevance for drawing policy and practice implications for Poland. This purposive selection aimed to provide a balanced and informative overview of international experiences rather than an exhaustive global comparison.

The search yielded 1505 records (1440 from databases and 65 from additional sources). After removal of 385 duplicates, 1120 records were screened by title and abstract, of which 940 were excluded. A total of 180 full-text articles were assessed for eligibility, and 56 met the inclusion criteria and were included in the final synthesis. This review provides a structured overview of international experiences and current evidence supporting the integration of pharmacogenetics into pharmacy practice, with particular attention to implications for the Polish healthcare system.

## 3. Results

### 3.1. Current Development of PGx Services

The development of genomics has helped to investigate the genetic basis of drug response variability. The identification of alleles responsible for rapid or slow metabolism of xenobiotics (any substance, typically a synthetic chemical, that is foreign to the body) has begun a new era in pharmacology. PGx is a combination of genetics and pharmacology, the aim of which is to tailor treatment to a specific patient, based on the analysis of their genome and gene polymorphism.

There are several mechanisms by which a drug can cause different effects on the body, the most important of them are:

- differences in drug metabolism that accelerate or delay its inactivation and elimination from the body;
- differences in the metabolism of the prodrug to the active form;
- genetic differences in drug transporters;
- target receptor mutations.

Gene polymorphism may affect the strength of the drug, its effectiveness, and toxicity. As many as 99.5% of the population have at least one polymorphism in a gene related to the metabolism of a drug available on the market [2,3]. In addition, pharmacogene polymorphisms are distributed differently in specific ethnic groups [4].

Currently, such gene polymorphism has a well-evaluated involvement in the treatment of:

- metabolic diseases (e.g., statins);
- anticoagulant treatment (warfarin, clopidogrel);
- psychiatry (antidepressants);
- oncology (molecular targeted therapy, fluoropyrimidines).

Building upon these insights, it is important to recognize that there are three main types of gene testing, each warranting separate consideration: single gene tests, genetic panel testing, and whole genome sequencing.

Given the significant influence of gene polymorphisms on individual responses to medications, genetic testing stands as a crucial safety measure in modern healthcare. By identifying a patient’s unique genetic makeup, clinicians can predict the likelihood of adverse drug reactions and tailor therapeutic choices accordingly. This proactive approach minimizes the risk of harm while optimizing the effectiveness of treatment, ensuring that therapies are not only safer but also genuinely beneficial for the patient’s underlying condition. In this way, PGx testing transforms the one-size-fits-all paradigm of drug therapy into a personalized strategy, fostering better health outcomes and greater patient safety.

Leading international organizations, such as the Clinical Pharmacogenetics Implementation Consortium (CPIC), the U.S. Food and Drug Administration (FDA), the Dutch Pharmacogenetics Working Group (DPWG), and the French Network of Pharmacogenetics (RNPGx), systematically review pharmacogenes and their polymorphisms. These bodies develop and disseminate evidence-based clinical guidelines to standardize the integration of PGx information into therapeutic decision-making and optimize patient outcomes.

The US-based Clinical Pharmacogenetics Implementation Consortium (CPIC) was founded in 2009, and its current evidence grading system using categories A–D has been in place at least since the early 2010s [5]. It defines itself as the international consortium of individual volunteers who are interested in facilitating use of PGx tests for patient care. The consortium divided drugs into several categories depending on the clinical significance of gene polymorphism:

- Category A (99 drugs)—genetic information should be used when making therapeutic decisions;
- Category B (32 drugs)—testing should be considered;
- Category C (271 drugs)—no recommendation, testing is available, but no confirmed clinical data;
- Category D (102 drugs)—testing is not recommended due to lack of data or test is not available [6].

The United States Food and Drug Administration (FDA), the regulatory authority overseeing pharmaceuticals, medical devices, cosmetics, and food products entering the U.S. market, maintains a comprehensive list of therapeutic agents containing pharmacogenomic information within their labeling. At present, this list encompasses 608 registered drugs [7].

For 62 of these medications, current scientific evidence supports the recommendation of PGx testing prior to initiation of therapy. Additionally, more than 60 drugs are identified for which genetic testing may provide potential clinical benefit [8].

Adverse drug reactions (ADRs) can result in missed workdays and discontinuation of therapy, posing serious risks to patient health by interrupting necessary treatment. More severe ADRs frequently lead to hospitalization and are responsible for approximately 5–10% of all hospital admissions [9,10]. These events not only threaten the wellbeing of individuals, but also impose a substantial financial burden on healthcare systems and state budgets. For this reason, integrating PGx testing into routine medical care is crucial—not only does it help safeguard patient health and safety, but it also has the potential to significantly reduce unnecessary healthcare spending linked to preventable ADRs.

WHO data indicates that only 50% of medications are taken properly, and one of the reasons for irregularities are side effects of medications [11]. According to the definition of pharmaceutical care, the therapeutic effect of a drug can only be achieved when the drug is used properly, i.e., adherence and compliance are followed precisely. Including pharmacies in PGx care tasks may be an effective way to avoid ADRs.

Pharmacists stand at the forefront of integrating PGx into healthcare, providing essential expertise in interpreting genetic test results and guiding safe, personalized treatment decisions.

Their advocacy for PGx adoption helps ensure that both healthcare systems and patients benefit from advances in precision medicine.

In Poland, community pharmacies are uniquely positioned to support the implementation of PGx. The Polish Pharmaceutical Act of 6 September 2001 tasks pharmacists with reporting ADRs, emphasizing their vital role in patient safety. Experiences from recent years, including the pandemic, have shown that pharmacies can serve as accessible centers for pharmaceutical care, promoting PGx awareness, supporting adherence to personalized therapies, and enhancing health outcomes across the population [12].

Genetic testing is poised to revolutionize primary care, representing a pivotal advancement in the transition toward personalized medicine. Decision makers who disregard these scientific advances risk missing opportunities to improve patient health, reduce costs, and stay at the forefront of medical innovation. Embracing this science is not only an investment in better care, but a necessity for leaders determined to advance with the times.

### 3.2. Situation in Selected Countries

North America and Western Europe are the main geographical regions of intense research and development in PGx. According to the Scopus database, as of 24 July 2023, Western European countries have published approximately 18,500 research items in this field, compared, for example, to approximately 15,500 items published in the United States [13].

The countries described in this article—Canada, USA, UK, France, Belgium, the Netherlands, and Spain—have demonstrated significant progress in the implementation of PGx into their healthcare systems, mainly through the presence of highly engaged organizations actively driving clinical integration. These nations have established initiatives, including national guidelines, dedicated research networks, and collaborative platforms, aimed at translating PGx research into routine medical practice. Consequently, they can serve as benchmarks for effective strategies in the implementation of PGx within healthcare infrastructures.

#### 3.2.1. Canada

The Canadian Pharmacogenomics Network for Drug Safety (CPNDS), established in 2005 as a Canada-wide, multidisciplinary research network established to identify genetic factors that influence drug efficacy and ADRs, plays a pivotal role in the advancement and implementation of PGx across Canada. A notable initiative, the “Genome Canada” project, entails the analysis of thousands of DNA samples, with the primary objective of enhancing the monitoring and prevention of ADRs. Particular focus has been placed on drugs frequently used in pediatric oncology and anticoagulant therapy [14].

The CPNDS aims to develop a comprehensive ADRs database, establish an educational platform, and facilitate the integration of PGx testing within national healthcare centers. Additionally, the organization is engaged in evaluating the broader impact of PGx on patients, their families, and the Canadian healthcare system, exemplified by the GE3LS (Genomics and its Ethical, Environmental, Economic, Legal, and Social Aspects) framework [15].

In the Canadian context, community pharmacies have begun to offer commercial PGx testing services; however, the availability of these services currently remains localized and has yet to achieve nationwide integration. Based on recent studies, pharmacies are ready to adopt PGx screening into practice and support integration of PGx on a national level [16].

#### 3.2.2. USA

The Pharmacogenomics Global Research Network (PGNR) based in the United States is an independent, global research network dedicated to advancing precision medicine by studying how an individual’s genetic makeup affects drug response; it aims to facilitate the implementation of PGx not only within national standards of care but also on a global scale. In addition to conducting research, PGNR is actively involved in lobbying and the formulation of strategic frameworks for healthcare systems, including cost estimations and analyses of potential savings for the budgets. PGNR members underscore the unique characteristics of the United States healthcare system, particularly the absence of a centralized, universally accessible system. Commercial insurers typically demonstrate reluctance to reimburse PGx testing costs. Consequently, the integration of PGx into clinical practice predominantly occurs at the local level, especially within major academic medical centers, and often remains limited to specific gene–drug pairs, such as clopidogrel–CYP2C19 [17,18].

At present, PGNR is actively involved in the implementation of gene–drug pair testing, including DPYD–fluoropyrimidine, clopidogrel–CYP2C19 in neurology, and tacrolimus–CYP3A5. In addition to these clinical integrations, the organization allocates funding for research grants and oversees ongoing clinical trials. Among these, two notable studies are GUARDD-US, which employs genetic testing in nephrology to elucidate gene disparities in specific diseases within the American population, and ADOPT-PGx, a project focused on the practical application of PGx in antidepressant and analgesic therapy. Reports detailing the outcomes of these investigations are anticipated in the near future [19,20].

There are several other pharmacogenomics and PGx initiatives in the US, the Implementing GeNomics In pracTicE Pragmatic Trials Network. (IGNITE PTN), the pilot CLIPMERGE PGx pilot study developed in Icahn School of Medicine at Mount Sinai, the eMERGE-PGx, The Imagenetics Initiative at Sanford Health, the 1200 Patients Project at the University of Chicago, and PREDICT panel testing at Vanderbilt University Medical Center, among others [21,22,23,24,25,26].

In terms of public access to PGx not via institutional programs, some of the US community pharmacy chains advertise their personalized medicine services by performing commercial PGx tests. Availability and reimbursement of direct-to-consumer PGx testing (DTC—tests that individuals can purchase and use independently without prescription or involvement from healthcare providers), limited training, limited access to prescribers, and limited access to patient medical records are considered barriers in the clinical delivery of PGx testing at pharmacies [27].

Lack of public awareness contributes to limited demand and slower adoption at the patient level. This gap emphasizes the importance of educational initiatives and outreach campaigns to ensure that the advantages of PGx reach broader populations and can be effectively implemented into routine pharmacy practice [28,29].

#### 3.2.3. United Kingdom

The United Kingdom prescribes over 1.1 billion medicines each year, with the annual medicines budget of the National Health Service (NHS, a government-funded healthcare system) reaching approximately £17.4 billion. Recognizing the clinical and economic burden of ADRs and suboptimal pharmacotherapy outcomes, the NHS has identified the integration of PGx into clinical practice as a promising strategy to improve therapeutic efficacy and safety [30].

A significant national effort, the “100,000 Genomes Project,” recently completed sequencing the genomes of 85,000 British patients diagnosed with rare and oncological diseases [31].

This initiative established a national genome bank designed to support research into the genetic basis of disease and inform personalized treatment approaches. Additionally, the UK Biobank has sequenced 500,000 genomes to date, further expanding the country’s genomic research infrastructure [32].

Following the completion of the “100,000 Genomes Project” in 2018, the Genomic Medicine Service was established to oversee the continued sequencing of genomes and facilitate the application of genomic knowledge to clinical research and practice. The NHS remains committed to funding genome sequencing projects and supporting the ongoing implementation of precision medicine strategies [33].

In 2022, a national report detailing the current status and future prospects of PGx in the UK was published [34].

The report highlighted that ADR-related hospitalizations cost the NHS an estimated £530 million per year, underscoring the potential of PGx implementation in routine medical practice to reduce these expenditures. In response, NHS England established a pharmacogenomics working group tasked with supervising the integration of PGx into the healthcare system. Currently, the NHS mandates genetic prescreening prior to initiating specific therapies, such as DPYD polymorphism testing before commencing fluoropyrimidine-based treatment [35]. To support the adoption of PGx, the pharmacy genomics workforce has been created, emphasizing the critical role of pharmacists in delivering PGx services [36]. Pharmacists, as prescribing professionals, are well positioned to manage PGx-driven care in community pharmacies, thus facilitating broader access to personalized medicine.

#### 3.2.4. France

In France, the integration of PGx testing into clinical practice is steadily advancing, particularly within specialized fields such as oncology, cardiology, and psychiatry.

The French Society of Pharmacology and Therapeutics (Société de Pharmacologie et de Thérapeutique, SPT) issues guidelines and publications on PGx testing for specific therapeutic areas, including thiopurines, clopidogrel, and oncology (such as KRAS mutations for targeted therapies and DPYD for fluoropyrimidines) [37]. Broader adoption of PGx continues, supported by ongoing research initiatives and pilot programs, with a primary focus on drugs that have well-established pharmacogenomic markers [38].

To support clinical decision-making, the French National Network of Pharmacogenetics (RNPGx), a network of experts in the field, provides comprehensive recommendations for PGx implementation. RNPGx assigns priority levels to its guidance based on the strength of available evidence and clinical utility (essential, advisable, possibly helpful test), underlining the need for systematic evaluation and consolidation of PGx research [39].

In France, more than 50 laboratories offered PGx testing several years ago, although these services were mostly limited to a small number of indications [40]. To fully unlock the potential of PGx, further steps must be taken to expand implementation, include community pharmacies in the process, increase validation efforts, and raise awareness and acceptance among healthcare providers [41].

#### 3.2.5. Belgium

Belgian expenditures on PGx reach €1.4 million per year. In outpatient settings, part of the PGx test is paid by the patient, part by the state. The Belgian government decided to cover the cost of 22 genetic tests (including CYP2C9 metabolized antidepressants therapy, clopidogrel, proton pump inhibitors; VKORC1 therapy with vitamin K antagonists). Currently, there are eight national centers that can order these tests as part of the state healthcare system. The oncological treatment constitutes more than half of the testing. Quantitatively, most tests are performed on the thiopurine–TPMT drug–gene pair. In 2024, a comprehensive report on the status of PGx in the country and around the world was published (KCE Report 382), detailing the state of knowledge and challenges of PGx, which proves how seriously the vision of implementing PGx on a large scale is taken in this country [42].

#### 3.2.6. The Netherlands

The Dutch Pharmacogenetic Working Group (DPWG) stands as an internationally recognized authority in PGx research. As a multidisciplinary panel, the DPWG develops and disseminates evidence-based guidelines to enhance pharmacotherapeutic decision-making. Their recommendations are widely cited in scientific literature and have contributed substantially to the international visibility and credibility of Dutch efforts in pharmacogenomics [43].

In addition to the DPWG, other scientific institutions in the Netherlands are actively engaged in PGx research. At the national level, Leiden University Medical Center (LUMC) conducts pivotal clinical trials in this field. While oncology remains a primary area of focus, LUMC’s research also includes the identification of novel biomarkers and the integration of pharmacogenomics into routine hospital clinical practice. Of note, the LUMC investigates the phenomenon of phenoconversion—the divergence between genetically predicted drug metabolism (genotype) and actual observed metabolism (phenotype). An ongoing trial, Personalized Therapeutics @LUMC (clinical trial no. NCT05508763), is evaluating a panel of 14 pharmacogenes in 2000 patients, with a 12-month observation period assessing the incidence of reported ADRs. This study is expected to conclude by October 2026 [44].

Furthermore, Dr. Marieke Coenen at University Medical Center Rotterdam has initiated the P4 Care study (Pharmacogenetic-passport for Optimizing Drug Treatment Before the First Pharmacological Treatment for Psychiatric Conditions in Primary Care). This project seeks to determine whether implementing a PGx passport can facilitate the selection of optimal drug therapies prior to the initiation of psychiatric pharmacological interventions in the primary care setting [45].

A pilot program conducted between 2019 and 2020 involved a network of 22 Dutch pharmacies, where 611 patient samples underwent PGx testing. The findings revealed pharmacogenetically relevant gene variants in 99.2% of the samples, with 90.9% of patients exhibiting more than one actionable pharmacogene. Test results were systematically communicated to both patients and their treating physicians. Post-project analysis identified several barriers to widespread implementation of PGx in community pharmacies: insufficient knowledge of PGx among healthcare professionals, absence of standardized procedures for relaying test results to patients, and the lack of clearly defined communication pathways between physicians and pharmacists. These findings underscore the need for comprehensive educational initiatives, improved clinical infrastructure, and the development of integrated communication systems to fully realize the potential of PGx in Dutch clinical practice [46].

#### 3.2.7. Spain

Spain represents a notable case in the implementation of PGx. The country’s healthcare system is characterized by its division into 17 autonomous communities and two autonomous cities, with implementation decisions predominantly made at the regional level. National coordination is facilitated by the Spanish Society of Pharmacogenetics and Pharmacogenomics (SEFF), established in 2005 to oversee PGx initiatives across Spain. Significant policy developments include the 2018 proposal by the Spanish Senate to launch a national program in genomics and personalized medicine, accompanied by a planned investment of €25 million to drive research efforts. In 2023, the Spanish Ministry of Health, in collaboration with a multidisciplinary expert panel, introduced a comprehensive national PGx catalog. This catalog primarily addresses the clinical domains of oncohematology, cardiology, ophthalmology, metabolic diseases, neurology, and psychiatry, and outlines genetic mutations relevant to 22 major drugs along with clinical indication criteria for PGx testing [47].

To date, clinical recommendation documents have been published for 12 gene–drug pairs (https://seff.es/), with additional publications forthcoming [48]. Furthermore, a publicly accessible database of PGx biomarkers has been established to support the implementation of personalized medicine strategies at the level of Spain’s autonomous regions [49].

An important organizational feature is that, in 42% of Spanish institutions engaging in PGx, hospital pharmacies are designated as the supervising entities for the testing process. This supervisory role underscores the integration of PGx within institutional clinical workflows and highlights the importance of hospital pharmacies in coordinating and standardizing PGx testing and interpretation [50].

#### 3.2.8. European Union

In 2016, the European Union initiated the Ubiquitous Pharmacogenomics (U-PGx) consortium to address critical challenges associated with the implementation of PGx across Europe. This consortium represents a collaborative endeavor, bringing together 16 organizations with expertise in the field [51]. One of the consortium’s key initiatives is the Pre-emptive Pharmacogenomic Testing for Preventing Adverse Drug Reactions (PREPARE) study, which constitutes the first large-scale, multicentric investigation into the clinical utility of pre-emptive PGx testing [52].

The PREPARE study evaluated a panel comprising approximately 40 allelic variants across 13 pharmacogenes pertinent to the therapeutic management of over 40 pharmaceutical agents. The study encompassed 8000 patients recruited from seven European countries (the Netherlands, Spain, United Kingdom, Italy, Austria, Greece, and Slovenia). The primary objectives included assessment of the feasibility, cost-effectiveness, and clinical benefit of pre-emptive PGx testing, as well as identification of barriers to widespread adoption within healthcare systems. In the study protocol, pre-emptive testing was defined as the simultaneous analysis of multiple gene–drug pairs prior to the need for pharmacotherapy, thereby equipping clinicians with readily accessible genetic information to inform prescribing decisions. The principal findings of the PREPARE study demonstrated a 30% reduction in clinically significant ADRs in the intervention cohort [53]. Nevertheless, several methodological limitations have been noted in subsequent analyses, including the absence of blinding, ambiguities in outcome assessment criteria, and a reduction in ADRs observed within control groups. These factors have prompted ongoing discussions regarding the interpretation and generalizability of the study results within the context of PGx implementation research.

Across the analyzed countries, several consistent patterns emerge. Countries with centralized healthcare systems and strong national coordination mechanisms, such as the United Kingdom and the Netherlands, demonstrate more advanced and systematic PGx implementation. In contrast, systems characterized by fragmented funding structures, such as the United States, tend to show localized and uneven adoption. The presence of dedicated national guidelines, integration with electronic health systems, and reimbursement mechanisms appear to be key enabling factors. Conversely, limited reimbursement, lack of interoperability, and insufficient professional training are consistently identified as major barriers across settings. These cross-country differences highlight that the success of PGx implementation is determined not only by scientific readiness, but primarily by system-level organization and policy support.

Table 1 highlights key differences in implementation maturity, reimbursement models, and the role of pharmacists, allowing for direct comparison between countries.

In Poland, the implementation of pharmacogenetics in community pharmacy remains at an early stage and is not yet supported by a national reimbursement framework. At present, pharmacogenetic testing is available mainly through commercial laboratories rather than as an integrated publicly funded pharmacy service. Examples include single-gene assays, such as CYP2C9 and CYP2C19 testing, as well as broader pharmacogenetic panels covering multiple clinically relevant genes. At the same time, the wider pharmacy sector in Poland has expanded its clinical competencies: under the current pharmaceutical care framework, pharmacists may provide consultations, perform selected diagnostic tests, and participate in vaccination services after completing dedicated training. Nevertheless, pharmaceutical care services are still not financed by the public payer, and the debate in Poland has focused primarily on how to introduce sustainable financing and implementation mechanisms. For this reason, although the legal and professional foundations for future PGx services are gradually emerging, practical implementation in community pharmacies still requires policy support, reimbursement pathways, and targeted education in pharmacogenetics.

### 3.3. Pharmacoeconomics—Current Evidence and International Experience

Pharmacoeconomics is a branch of health economics that systematically examines the value of one pharmaceutical drug or drug therapy compared to another. Its primary objectives are to inform decision-making processes regarding the allocation of scarce healthcare resources, evaluate the cost-effectiveness of therapeutic interventions, and enhance the quality and efficiency of patient care. By integrating both economic and clinical outcomes, pharmacoeconomic analyses provide essential insight into the real-world impact of pharmaceutical innovation, especially as healthcare systems confront rising expenditures and the need for personalized medicine.

The increasing integration of PGx into clinical practice has prompted considerable interest in its economic implications. However, the broad adoption of PGx is contingent not only upon clinical utility but also upon rigorous assessments of its cost-effectiveness in diverse healthcare settings.

There are several publications on PGx cost analysis in psychiatry, cardiology, oncology, and HIV therapy. For example, in Canada, research has examined the potential impact of pharmacogenomic-guided care on government healthcare expenditures [54]. Estimates suggest that preemptive genetic testing before prescribing antidepressants could yield savings ranging from C$1035 to C$4926 per patient, with cumulative savings potentially reaching C$956 million. Health benefits were quantified at 0.064 life years and 0.381 quality-adjusted life years (QALYs) per patient, translating to 12,436 life years and a total of 74,023 QALYs over a 20-year horizon. These benefits are primarily attributed to the reduction or prevention of treatment failure and the development of treatment-resistant depression, with projections indicating a 37% decrease in the number of patients experiencing treatment-resistant depression within two decades. Sensitivity analyses indicate that the initial investment in PGx testing could be recouped within approximately two years of implementation.

In 2022, Morriss et al. conducted a systematic review of PGx economics [55]. In total, 108 studies were included, of which 44% rated PGx as cost-effective, 27% cost-saving, 20% not cost-effective, and 9% uncertain [56]. Both reviews highlight the lack of sufficient data to draw reliable conclusions. The limitations of the analyzed studies include calculations based on hypothetical populations, the absence of randomized, blinded studies, population differences in gene polymorphisms, and local differences in procedure costs. The most robust data exist for clopidogrel, for which the majority of studies confirm cost-effectiveness. However, the extrapolation of these findings to entire healthcare systems or other country settings remains limited.

Pharmacogenetic variants that affect drug response are extremely common worldwide, but their frequencies differ strongly by gene and population. Across many large and ethnically diverse cohorts, actionable pharmacogenetic polymorphisms are nearly universal, with most people carrying multiple high-impact variants. Genes such as CYP2D6, CYP2C19, CYP2C9, CYP3A5, VKORC1, DPYD, TPMT, NUDT15, SLCO1B1, ABCG2, NAT2, G6PD, and specific HLA-B alleles show the highest prevalence and clinical relevance, though exact frequencies are strongly population-specific. In large population cohorts, >99% of individuals carry at least one actionable pharmacogenetic variant, e.g., UK Biobank (487k people): 99.5% had ≥1 actionable variant [2], average atypical response predicted for ~10–12 drugs; Danish cohort (77k): >99.9% had ≥1 divergent phenotype, 87% had ≥3 actionable variants [57]; Swiss hospital + population cohorts: 97.3% had ≥1 actionable variant [58]; Sardinians: 99.43% predicted atypical response to ≥1 drug, average ~17 drugs per person [59]; Saudi and Qatari cohorts: 99.2% and 99.5% carried ≥1 actionable genotype [3,4].

A persistent challenge for authors of such analyses is the reliable estimation of hospitalization costs attributable to ADRs [60].

In 2022, a cost-effectiveness assessment of twelve-month clopidogrel therapy compared with genotyping and alternative drug therapy was performed in Poland [61]. The results did not demonstrate the cost-effectiveness of PGx, largely influenced by the high market cost of tests in Poland, itself a result of limited reimbursement and low uptake. Additionally, alternative treatments to clopidogrel are more expensive. Nevertheless, the authors reference other studies that have confirmed cost-effectiveness under different market conditions.

Although some analyses are available, including local economic evaluations, the evidence remains limited and context-specific, which restricts its generalizability for system-level decision making. There is a clear need for new, high-quality research to generate robust evidence, a need heightened by rapid economic changes related to inflation and fluctuating costs. Only through well-designed and comprehensive studies can policymakers and healthcare providers make informed, evidence-based decisions regarding the allocation of resources for PGx testing in the Polish context.

Depending on the gene tested, the cost of PGx tests varies between $100 and $500. In Poland, commercial PGx tests are available for drugs in cardiology, metabolic diseases, oncology, and neurology, including clopidogrel, warfarin, captopril, simvastatin, rosiglitazone, risperidone, corticosteroids, tacrolimus, thalidomide, allopurinol, carbamazepine, and phenytoin, among others. Panel (multi-gene) testing may help reduce these costs, and such panels are now offered by Polish commercial laboratories. Advances in laboratory technology also promise further cost reductions; for example, a study in China demonstrated that the cost per test could be reduced to a few US dollars when screening gene panels in large cohorts (e.g., 22,918 individuals) [62].

Creating a genome bank, as underway in the United Kingdom, represents a potential future direction. This approach would obviate the need for repeated blood draws during therapy implementation, as PGx data could be retrieved directly from the bank, thereby avoiding delays [63]. However, this solution raises considerable legal and ethical concerns, including the handling of sensitive data that may impact not only individuals but also their relatives across generations. Observing the implementation of such projects in the UK may provide important insights for managing future challenges. A major limitation is the cost—Britain’s 100,000 Genomics Project, for example, required a £300 million investment, though innovations in sequencing may significantly reduce future costs.

As the NHS report indicates [64], the implementation of PGx into routine clinical practice will not be feasible without commercial financing. In the UK, more than £3.3 billion has been raised for genomic research from over 50 commercial companies, suggesting strong industry interest in the future of PGx.

Examples of reimbursed PGx tests in European countries such as the UK, France, and Switzerland include: abacavir-HLA-B5701, azathioprine-TPMT, mercaptopurine-TPMT, capecitabine-DPYD, fluorouracil-DPYD, irinotecan-UGT1A1, and carbamazepine-HLA-A3101 or HLA-B1502 [65].

The variability in pharmacoeconomic results across countries is driven by several interrelated factors. First, the price of PGx tests differs substantially between healthcare systems depending on laboratory infrastructure, testing volume, and reimbursement arrangements. Second, the cost-effectiveness of PGx depends on the clinical context, including the prevalence of actionable gene variants in the population, the baseline risk of adverse drug reactions, and the availability and cost of alternative therapies. Third, regional differences in healthcare organization, hospitalization costs, and public funding models strongly influence whether the economic benefits of PGx can be realized in practice. As a result, findings from one country cannot be directly transferred to another without considering local pricing, prescribing patterns, and payer perspectives. In countries where PGx has shown more favorable economic outcomes, implementation has usually been supported by broader reimbursement mechanisms, higher testing volumes, and integration into routine care pathways.

The cost-effectiveness of pharmacogenetic testing is highly context-dependent and influenced by several key factors. Evidence suggests that PGx is more likely to be cost-effective in settings where there is a high prevalence of actionable gene variants, a significant baseline risk of adverse drug reactions, and the availability of clinically relevant alternative therapies. Economic benefits are also more evident when testing is applied pre-emptively or at scale, allowing results to inform multiple treatment decisions over time. In contrast, cost-effectiveness may be limited in systems with high testing costs, low integration into clinical workflows, or lack of reimbursement mechanisms. Importantly, the value of PGx is closely linked to healthcare system organization, including access to electronic health records, clinical decision support systems, and interprofessional collaboration. These findings indicate that economic outcomes cannot be generalized across countries and must be interpreted within specific healthcare and policy contexts.

### 3.4. Challenges and Opportunities in the Implementation of PGx: A Scientific Perspective

A significant barrier to the integration of pharmacogenetics into routine clinical practice is the insufficient knowledge among healthcare professionals. Enhanced education would not only mitigate the incidence of ADRs, but also reduce risks associated with polypharmacy, frequency of medical visits, work absenteeism, and hospitalizations. However, accessibility and the cost of PGx tests—as well as the absence of reimbursement—remain persistent obstacles. Except in the context of oncology and targeted molecular therapies, most test costs are not covered by private insurance providers.

Standardization of testing methodologies, as well as improvements in sensitivity and specificity, are critical challenges in the broader adoption of PGx. The presence of rare alleles, highly polymorphic gene regions, pseudogenes, and extragenic variations further complicate test interpretation. Moreover, the clinical utility of some PGx tests is limited by the low prevalence of specific genotypes in certain populations [66].

The reduction of turnaround time for PGx test results is essential for clinical utility, emphasizing the importance of point-of-care testing—either at the patient’s bedside (“bedside testing”) or the pharmacy counter [67]. “Bedside testing” stands for a diagnostic or laboratory testing performed directly at the patient’s location, typically in a clinical setting such as a hospital room, outpatient clinic, or even in emergency situations. This solution is important as patients cannot, or refuse to, wait days or weeks for test results before commencing the prescription. Point-of-care diagnostics facilitate rapid clinical decision-making, particularly in settings where immediate drug administration is required (e.g., clopidogrel prior to percutaneous coronary intervention). Additionally, PGx test results must be presented in a manner that is both accessible and easily interpretable by clinicians.

Given the ongoing evolution of PGx research, clinical recommendations necessitate continuous re-evaluation. It is imperative to maintain reliable, easily accessible, and frequently updated sources of information for healthcare professionals. Currently, there are no unified international guidelines; differences exist between major recommendations (e.g., CPIC and DPWG), stemming from variations in methodology, timing of evidence review, and national clinical standards [68]. Notably, several major scientific societies, including the American Heart Association and the American College of Cardiology Foundation, do not currently endorse routine PGx testing before pharmacotherapy initiation, reflecting ongoing debate within the field.

Incorporating PGx recommendations into treatment guidelines requires collaboration among legislators, regulatory agencies, and scientific societies. Regulatory frameworks (e.g., EMA, FDA, Health Canada) now require that PGx information impacting drug safety and efficacy be included in drug registration documentation [66,69,70]. Innovative clinical decision support system (CDSS) tools, such as FARMAPRICE and others, have been developed to facilitate the integration of PGx data into clinical workflows [71].

The role of pharmacists in PGx implementation is increasingly recognized. Pharmacists, as the final point of contact before therapy initiation, are optimally positioned to verify the appropriateness of pharmacotherapy in relation to a patient’s genetic profile. In Poland, for example, community pharmacies serve approximately two million patients daily. The COVID-19 pandemic demonstrated the adaptability of pharmacists in expanding their clinical role [71]. Given their accessibility, integrating PGx services into community pharmacy practice is justified, though it requires collaborative efforts among physicians, pharmacists, and laboratories [72].

Legislative action is needed to leverage pharmacists’ expertise and initiate PGx testing at the community pharmacy level. The inclusion of PGx within pharmacy and medical education is crucial to equipping future healthcare professionals with the competencies necessary for precision medicine. Targeted training programs for clinicians and pharmacists should precede broader implementation, ensuring proficiency in test ordering, interpretation, and application. Establishing support systems—such as online consultation platforms and dedicated, regularly updated databases—would facilitate integration and ongoing education.

Pilot programs, as pioneered in the Netherlands, could serve as a valuable model for evaluating practical aspects, feasibility, barriers, and acceptance of PGx solutions prior to national-scale implementation. Such initiatives, combined with robust cost-effectiveness analysis, would inform policy decisions and resource allocation.

Ultimately, a collaborative, education-driven approach that empowers pharmacists will be key to realizing the potential of PGx in personalized medicine. Overview of pharmacogenetic services in selected countries is presented in the Table 2.

### 3.5. Pharmacoepigenetics and Phenoconversion

From a clinical implementation perspective, understanding phenoconversion and pharmacoepigenetic mechanisms is essential, as these factors may influence the real-world applicability and interpretation of pharmacogenetic (PGx) results.

Drug response is shaped by epigenomic regulation of ADME genes; this can produce phenoconversion, a divergence between genotype predicted and actual metabolic phenotype [75,76]. Mechanistic example: DNA methylation within CYP3A4 regulatory regions attenuates pregnane X receptor–mediated transactivation and blunts induction, reducing clearance of CYP3A4 substrates despite a “normal” genotype [77]. Clinical signal: methylation of donor hepatic ABCB1 correlates with tacrolimus blood concentrations in liver-transplant recipients [78]. Environmental leverage: cadmium increases ABCB1/P gp expression in human renal proximal tubule cells, and prenatal Cd exposure leaves persistent epigenetic marks in placenta and offspring [79,80]. Lead exposure generates stable blood DNA methylation signatures that track cumulative body burden and associate with neurological outcomes, offering scalable exposure biomarkers [81,82]. Operationally, PGx results should be interpreted alongside phenoconversion drivers (concomitant inhibitors/inducers, inflammation, age, environmental exposures) and, where feasible, simple epigenetic biomarkers [78].

From a practical perspective, phenoconversion and epigenetic factors have important implications for pharmacy practice. Pharmacists can incorporate awareness of phenoconversion driver, such as concomitant medications, inflammatory status, age, and environmental exposures, when interpreting PGx results and providing patient counseling. This approach allows for more accurate assessment of actual drug metabolism beyond genotype alone. In the future, simplified phenotype assessments or clinically applicable epigenetic biomarkers may further support pharmacists in optimizing therapy, although their routine use in community pharmacy settings remains limited at present.

These considerations highlight that effective implementation of PGx in clinical and community pharmacy settings requires not only access to genetic data, but also the ability to interpret such data in the context of dynamic, non-genetic modifiers of drug response.

## 4. Discussion

The implementation of pharmacogenetics (PGx) in community pharmacy practice represents one of the most significant frontiers in modern pharmaceutical care. This review demonstrates that despite clear scientific justification and strong evidence of clinical utility, the pace of PGx adoption remains uneven across healthcare systems. Differences in regulation, reimbursement, and professional education largely explain the variability in implementation observed between countries.

North American and Western European nations have made the most substantial progress, largely due to early establishment of national networks such as the CPIC, DPWG, CPNDS, and RNPGx. Their structured guidelines, integration of pharmacogenomic data into clinical decision support systems (CDSS), and consistent educational efforts have enabled translation of genomic discoveries into patient care. In contrast, in many Central and Eastern European countries—including Poland—implementation remains limited to isolated research projects and commercial offerings, lacking the systemic integration and reimbursement mechanisms necessary for large-scale impact.

The role of pharmacists is particularly prominent in this context. As accessible healthcare professionals with expertise in pharmacotherapy, pharmacists are ideally positioned to interpret PGx test results, educate patients, and collaborate with prescribers to ensure optimal therapeutic outcomes. Experiences from the Netherlands, the United Kingdom, and Canada clearly indicate that when pharmacists are adequately trained and supported by policy frameworks, they can effectively facilitate PGx-guided therapy. However, barriers such as limited access to clinical data, insufficient awareness among prescribers, and lack of reimbursement for pharmacist-led PGx consultations hinder broader adoption.

A comparative perspective indicates that successful implementation of PGx is consistently associated with several system-level factors. These include the existence of national strategies or coordinated programs, integration of PGx into clinical decision support systems, availability of reimbursement pathways, and clearly defined roles for healthcare professionals, particularly pharmacists. In contrast, implementation remains limited in settings where PGx is restricted to research initiatives or commercial testing without integration into routine care. The lack of access to patient data, unclear professional responsibilities, and insufficient interprofessional collaboration further hinder translation into practice. These findings suggest that PGx implementation should be approached primarily as a health system innovation rather than solely a technological advancement.

Despite the growing recognition of pharmacists’ role in PGx implementation, several pharmacist-specific barriers remain insufficiently addressed. One of the key challenges is limited access to patient clinical and genetic data, particularly in healthcare systems where electronic health records are fragmented or not fully accessible to community pharmacists. This restricts the ability to interpret PGx results in a clinically meaningful context. In addition, concerns related to professional liability and unclear scope of responsibility may discourage pharmacists from actively engaging in PGx-guided decision-making, especially in settings where formal guidelines or legal frameworks are lacking. Time constraints, lack of reimbursement for cognitive services, and insufficient integration with prescribing systems further limit the feasibility of implementing PGx in routine community pharmacy practice. Addressing these barriers will require not only educational interventions, but also system-level solutions ensuring data access, clear professional roles, and financial incentives.

From a Polish legal perspective, the implementation of pharmacogenetics in community pharmacy raises additional challenges related to the practical application of GDPR regulations. Genetic data are classified as a special category of personal data, and their processing requires a clear legal basis, appropriate safeguards, and strict adherence to data minimization principles. In practice, this creates barriers to efficient data sharing between laboratories, physicians, and community pharmacists, particularly in the absence of fully integrated electronic health record systems. Pharmacists often do not have direct access to patients’ genetic test results unless these are actively provided by the patient or integrated into shared medical documentation. Furthermore, uncertainty regarding data responsibility and the scope of professional access to genetic information may limit pharmacist involvement in PGx-guided care. Addressing these issues will require the development of interoperable data systems, clear regulatory guidance on data sharing, and defined roles for pharmacists within the framework of genetic data processing in healthcare.

Real-world studies highlight the feasibility of pharmacogenetics in community pharmacy. A Dutch pilot showed that PGx testing identified clinically actionable variants in most patients and often led to therapy adjustments, while confirming the feasibility of pharmacist-led result interpretation. Similarly, the Canadian ICANPIC study demonstrated that pharmacists can effectively deliver PGx screening and support medication management, including identifying patients at risk of adverse drug reactions. Although large-scale outcome data remain limited, these findings provide proof-of-concept that PGx services can be successfully implemented in community pharmacy practice.

Importantly, emerging evidence from community pharmacy settings suggests that pharmacogenetic services may translate into clinically meaningful outcomes, including improved medication safety through the identification and prevention of potential adverse drug reactions, more appropriate drug and dose selection, and enhanced patient engagement in therapy. Pharmacist-led PGx interventions have also been associated with improved medication understanding and adherence-related behaviors, reflecting the added value of personalized counseling. While these findings indicate a promising impact on patient-level outcomes, robust evidence demonstrating reductions in hard clinical endpoints, such as hospitalizations or ADR-related admissions, remains limited and warrants further investigation in large-scale, real-world studies.

The cost-effectiveness of PGx testing remains a central issue. Although studies in Canada, the United Kingdom, and the United States demonstrate potential economic benefits—particularly through prevention of adverse drug reactions (ADRs) and treatment failures—these findings are not yet universally reproducible. Many existing analyses rely on modeling rather than real-world data, and they are sensitive to local healthcare costs and testing prices. In Poland, for instance, the absence of reimbursement and the high cost of commercial PGx testing significantly limit feasibility. Future research should therefore prioritize pragmatic trials evaluating real-world economic and clinical outcomes, focusing on cost-sharing mechanisms between healthcare systems and private laboratories.

Despite growing interest in PGx, the underlying evidence base remains heterogeneous and, in many areas, still limited. A substantial proportion of available data is derived from pilot studies, observational analyses, or model-based economic evaluations rather than large-scale randomized trials. Moreover, variability in study design, population characteristics, and outcome definitions complicates direct comparison across settings. As a result, while the clinical rationale for PGx is well established, the strength of evidence supporting its routine implementation—particularly in community pharmacy—remains uneven and context-dependent.

Ethical and legal aspects also warrant attention. Genomic data constitute highly sensitive information that may impact not only individual patients but also their families. Data protection, informed consent, and secure storage systems must be guaranteed to maintain public trust and comply with GDPR regulations in the European Union. Lessons learned from large genomic programs in the United Kingdom and the European Union’s PREPARE project could inform national strategies for safe and equitable PGx implementation in Poland and beyond.

According to Article 4(13) of Regulation (EU) 2016/679 of the European Parliament and of the Council of 27 April 2016 on the protection of natural persons with regard to the processing of personal data and on the free movement of such data, and repealing Directive 95/46/EC (General Data Protection Regulation)—hereinafter “GDPR”—“genetic data” [83] means personal data relating to the inherited or acquired genetic characteristics of a natural person which give unique information about the physiology or the health of that natural person and which result, in particular, from an analysis of a biological sample from the natural person in question [84].

At the same time, Recital 34 GDPR indicates that: “Genetic data should be defined as personal data relating to the inherited or acquired genetic characteristics of a natural person, obtained from the analysis of a biological sample of that natural person, in particular from the analysis of chromosomes, deoxyribonucleic acid (DNA) or ribonucleic acid (RNA), or from the analysis of other elements enabling the obtaining of equivalent information.”

These data have been classified as a special category of sensitive data and, pursuant to Article 9(1) GDPR, are subject to a general prohibition on their processing. However, paragraph 2 of that provision lays down a number of exceptions. In particular, it should be noted here that the processing of such data is permitted for the purposes of preventive or occupational medicine, medical diagnosis, the provision of health or social care, treatment or the management of health or social care systems and services (Article 9(2)(h) GDPR). Moreover, Article 9(2)(j) GDPR provides for an exception allowing the processing of personal data, inter alia, for scientific research purposes. In each of the cases referred to in Article 9(2)(h), (j) GDPR, the processing of such data must take place in accordance with Union law or Member State law. At the same time, the processing of such data is permissible by, or under the responsibility of, a person who is subject to a legal obligation of professional secrecy (Article 9(3) GDPR).

It should be noted that the GDPR does not contain a legal definition of “scientific research”. However, Recital 159 GDPR refers to a broad understanding of the term, covering, inter alia, technological development and demonstration, fundamental research, applied research, and privately funded research. It should nevertheless be emphasized that the European Data Protection Board recommends a cautious approach to expanding the definition of scientific research. Consequently, only activities that comply with the relevant sectoral methodological and ethical standards and good practices should be classified as scientific research.

Article 5 GDPR sets out the principles relating to the processing of personal data. In the context of sensitive data whose processing is permitted on the basis of the exceptions indicated above, particular emphasis should be placed on the principle of data minimization (point (c)), according to which the data must be adequate, relevant, and limited to what is necessary in relation to the purposes for which they are processed [82]. Among the principles relating to the processing of personal data, reference should also be made to the principle set out in Article 5(1)(e), according to which data should be kept in a form which permits the identification of data subjects. This condition will be fulfilled once effective anonymization of genetic data has been carried out (i.e., once they have been deprived of their identifying potential). This means that the information on the physiology or health of natural persons obtained as a result of anonymization will cease to constitute personal data. In practice, however, achieving a state of absolute anonymization of genetic data may prove extremely difficult, due to the constantly expanding scope of the processing of genetic information in cyberspace with the use of modern bioinformatics tools supported by cloud computing technologies [84,85].

## 5. Conclusions

Pharmacogenetics (PGx) represents a key component of the transition toward personalized medicine and offers a promising opportunity to enhance the safety and effectiveness of pharmacotherapy. Community pharmacists, as highly accessible healthcare professionals, are well positioned to support the implementation of PGx services, particularly in the areas of patient education, medication review, and therapy optimization. However, despite growing international experience, the integration of PGx into community pharmacy practice remains uneven and faces systemic barriers, including limited reimbursement, insufficient training, and restricted access to patient data.

To support effective implementation of PGx in community pharmacy practice, several actionable policy and practice recommendations can be identified:

- Establish reimbursement models for PGx testing and pharmacist-led cognitive services to ensure financial sustainability and wider access.
- Integrate PGx data into electronic health records (EHRs) with pharmacist access, enabling clinically meaningful interpretation and interprofessional collaboration.
- Develop and implement standardized education and training programs in PGx for pharmacists and other healthcare professionals.
- Introduce pilot programs in community pharmacies to evaluate feasibility, clinical outcomes, and cost-effectiveness in real-world settings.
- Create clear regulatory and clinical guidelines defining the role of pharmacists in PGx service delivery, including responsibilities related to data interpretation and patient counseling.

A coordinated, system-level approach that combines policy support, education, and clinical integration is essential to fully realize the potential of PGx in community pharmacy and to translate precision medicine into routine patient care.

## Figures and Tables

**Table 1:** Comparative overview of pharmacogenetics implementation in selected countries with relevance to community pharmacy.

Country	Key PGx Initiatives	Reimbursement Status	Role of Pharmacists (incl. Community Pharmacy)	Implementation Maturity	Key Lessons for Poland
USA	CPIC guidelines, PGNR, IGNITE, multiple academic programs	Partial, dependent on insurers	Limited in community pharmacies; mainly institutional settings	Fragmented, local	Need for reimbursement models and integration with EHR
Canada	CPNDS, Genome Canada, ICANPIC study	Partial (provincial systems)	Community pharmacies piloting PGx services	Moderate	Feasibility of pharmacy-based PGx screening
United Kingdom	NHS Genomic Medicine Service, 100,000 Genomes Project	Increasing reimbursement via NHS	Expanding role; planned integration into community pharmacy	Advanced (centralized)	Importance of national coordination and funding
France	RNPGx, SPT guidelines	Limited, mainly hospital-based	Minimal involvement of community pharmacies	Moderate	Need for broader system integration and awareness
Belgium	National reimbursement for selected tests (RIZIV-INAMI), KCE report	Partial, selected tests reimbursed	Limited data on pharmacy involvement	Moderate	Importance of national reimbursement lists
Netherlands	DPWG guidelines, LUMC research, pharmacy pilot programs	Increasing, mainly hospital-based	Active involvement of community pharmacies (pilot programs)	Advanced	Strong model for pharmacist-led implementation
Spain	SEFF, national PGx catalog (2023)	Increasing, region-dependent	Mainly hospital pharmacy supervision	Moderate	Role of national coordination with regional flexibility
Poland	No national program; commercial testing available	Limited, mostly out-of-pocket	No formal role in PGx services	Early stage	Need for policy, reimbursement, and education

PGx–pharmacogenetics; ADRs–adverse drug reactions; EHR–electronic health records. Implementation maturity reflects the level of integration of PGx into routine clinical practice (early–minimal or pilot-level implementation; moderate–partial integration; advanced–system-level implementation; fragmented–local or non-systematic implementation). Reimbursement refers to public or insurance-based coverage of PGx testing.

**Table 2:** Overview of pharmacogenetic services in selected countries *.

Country	Indications	Gene–Drug Pairs Monitoring *	Testing Local/Central	Cost Analysis Studies Available	Tests Available in Community Pharmacies	Reimbursement
USA	Oncology, cardiology, anti-inflammatory and analgesic drugs, neurology, psychiatry, immunology, gastroenterology, metabolic diseases, infectious diseases	608 drugs on the FDA list	Local	Yes	No	Reimbursed by insurers under certain conditions
Canada	As above	185 tests on the Health Canada list	Local	Yes	Yes	Some tests reimbursed by provincial healthcare systems
United Kingdom	As above	routinely or at the implementation stage: azathioprine, abacavir, fluoropyrimidines, carbamazepine, aminoglycoside antibiotics, clopidogrel	Central	Yes	Yes (currently at the stage of plans to implement the service in pharmacies by the NHS)	Coverage of tests costs is expanding, especially when supported by evidence and guidelines from NICE ** or other professional bodies
France	As above	Individual drug–gene pairs recommendation available on ClinPGx webpage, recently for mavacamten–CYP2C19 [73,74]	Local	No	No	Reimbursement levels vary and are limited to hospital settings; some tests may require prior authorization
Belgium	As above	The National Institute for Health and Disability Insurance (RIZIV-INAMI) genetic tests list—approximately 20 of them are PGx tests	Central	Analysis of international pharmacoeconomic studies in the KCE Report, May 2024	No data	Coverage for PGx tests is increasing, limited to hospital settings
Netherlands	As above	65 gene–drug pairs on the DPWG list	Local	Yes	Yes	Coverage for PGx tests is increasing, limited to hospital settings
Spain	As above	22 drugs on the Ministry of Health list	Local and Central	Yes	Hospital pharmacies	Coverage for PGx tests is increasing, limited to hospital settings
Poland	Commercial tests: oncology, cardiology, neurology, psychiatry, metabolic diseases, infectious diseases, anti-inflammatory and pain medications	No	No	No	No	coverage is not widespread, limited to hospital settings

* molecular targeted pharmacotherapy not included; data reviewed in August 2025. ** National Institute for Health and Care Excellence. PGx–pharmacogenetics; ADRs–adverse drug reactions; DTC–direct-to-consumer testing; NHS–National Health Service; NICE–National Institute for Health and Care Excellence. “Testing local/central” refers to whether PGx testing is performed at decentralized (local) or coordinated (central/national) levels. “Tests available in community pharmacies” indicates whether PGx services are directly accessible at the community pharmacy level. Reimbursement refers to public or insurance-based funding of PGx testing and may vary by indication and region.

## Data Availability

The data will available in corresponding author.
